# Supplementary material for: PolyFold: An interactive visual simulator for distance-based protein folding
Source: PLoS One. 2020 Dec 3;15(12):e0243331. doi: 10.1371/journal.pone.0243331 (PMC7714222; doi:10.1371/journal.pone.0243331)
Supplement: S1 Text — (PDF) [file pone.0243331.s001.pdf]

PolyFold: Protein Folding Simulator

Version 1.0.0, 2020

Andrew J. McGehee et al.
Department of Computer Science and Software Engineering
Auburn University
Auburn, AL

Correspondence: bhattacharyad@auburn.edu

Contents

1 Introduction ... **1**
1.1 A Letter from the Developer ... 1
1.2 What Is PolyFold? ... 1

2 Installation & License ... **3**
2.1 Dependencies ... 3
2.2 Downloading PolyFold ... 3
2.3 Installing PolyFold ... 4
2.3.1 Pre-built Packages ... 4
2.3.2 Building from Source ... 4
2.4 License ... 6

3 Features ... **7**
3.1 Protein Visualization & Manipulation ... 7
3.2 History & Save States ... 9
3.3 Optimization ... 10
3.3.1 Batch Gradient Descent ... 10
3.3.2 Simulated Annealing ... 12
3.4 Performance ... 14
3.5 Menus ... 14
3.6 Toolbar ... 16

4 Usage ... **19**
4.1 File Formats ... 19
4.2 Keyboard Shortcuts ... 20

5 Modifying PolyFold ... **21**

6 Acknowledgments ... **23**

Bibliography ... **24**

1 Introduction

1.1 A Letter from the Developer

First and foremost, thank you.

Thank you for downloading PolyFold. I began this project as a curious sophomore who knew nothing about protein folding, computational biology, or application development for that matter. I was deeply under-qualified. To have anyone download something that I built is a truly surreal feeling.

Thank you for taking the time to read this documentation. A lot of care and time went into building PolyFold and writing this manual. It is my great hope that PolyFold will be a useful tool for you which broadens your visual understanding of protein folding. I believe that as you tinker with PolyFold's features, you will discover like I did how vastly complex and interesting the protein folding problem is, and you will appreciate just how much collaborative math and science is needed in order to make progress in solving it. I have tried my very best to make PolyFold sleek, intuitive, minimal, and fun to use. I have also tried to write this manual in plain English, providing visuals where words fall short. I've attempted to be exhaustive and thorough. If PolyFold can do it, it should be somewhere in this manual.

Finally, thank you in advance for helping me improve. As you read this document and as you use PolyFold I ask that you pay attention to your feelings and your experience. If something doesn't make sense in the manual, if PolyFold has a bug, if you want to see a new feature, please let me know. You can file issues on PolyFold's GitHub repository at

https://github.com/Bhattacharya-Lab/PolyFold

Always learning,

-Andrew

1.2 What Is PolyFold?

PolyFold is an interactive, visual simulator for distance-based protein folding. The goal of PolyFold is to give both researchers and non-experts an intuitive and easy-to-use visual interface which elucidates the *process* of distance-based protein folding. PolyFold embeds powerful stochastic optimization algorithms with on-demand customizations and interactive manipulations in real-time to fold a protein molecule through the satisfaction of spatial constraints derived from a protein's inter-residue distance matrix. It is the hope of the PolyFold team that PolyFold will help fill out its user's intuition of the

processes of distance-based protein folding. No prior scientific background is needed to work with PolyFold!

The name PolyFold is inspired by the idea that the protein folding problem must be tackled as a unified community of both academic and citizen scientists spanning diverse fields of science, technology, engineering, and mathematics.

2 Installation & License

2.1 Dependencies

PolyFold was developed in Java and is designed for long term support. Accordingly, PolyFold depends on the Java 11 JDK, which can be downloaded from

https://oracle.com/technetwork/java/javase/downloads/#JDK11

PolyFold also depends on the JavaFX 11 API, which can be downloaded at

http://gluonhq.com/products/javafx/

PolyFold requires two environment variables to be set in order for it to run properly. These are the `JAVA_HOME` and `JAVAFX_HOME` variables. The `JAVA_HOME` variable needs to point to your installation of Java 11. More specifically, it needs to point to the parent directory of your Java 11 installation's `bin` directory. The `JAVAFX_HOME` variable needs to point to your JavaFX installation. More specifically, it points to the `lib` directory within your JavaFX installation. On my Mac, these variables happen to be exported as:

```
export JAVA_HOME=/Library/Java/JVM/jdk-11.0.5.jdk/Contents/Home
export JAVAFX_HOME=/Library/Java/JavaFX/javafx-sdk-11.0.2/lib
```

On my Debian machine, these directories happen to be:

```
export JAVA_HOME=/usr/lib/jvm/jdk-11.0.5
export JAVAFX_HOME=/usr/lib/jvm/javafx-sdk-11.0.2/lib
```

Please note that the above directories will likely be different on your own machine.

2.2 Downloading PolyFold

PolyFold is open source and hosted on GitHub. It is distributed as three pre-built packages, one for Mac OS 10.11+, one for Windows 10 64 bit, and one for the stable release of Debian or Ubuntu Linux. Both the source code and the OS dependent packages can be downloaded at:

https://github.com/Bhattacharya-Lab/PolyFold

2.3 Installing PolyFold

2.3.1 Pre-built Packages

Mac OS

First, clone the GitHub repository. Open the `Mac` directory. Unzip the `PolyFold.tgz` tar ball either by double clicking it or using the command line. Move the unzipped PolyFold application into your Applications folder.

Debian / Ubuntu

First, clone the GitHub repository. Open the `Linux` directory. Make sure that the `installer` file has execution permissions. Run `./installer`. After installation, it is safe to remove the cloned repository.

Windows

First, clone the GitHub repository. Open the `Windows` directory. Drag and drop the PolyFold EXE file into the location of your choice.

2.3.2 Building from Source

The recommended method for installing PolyFold from source is to use the Maven build automation tool.

Mac OS

1. If you do not have homebrew installed, you may install it following the instructions at https://brew.sh/
2. Install Maven

   ```
   $ brew install maven
   ```

3. Export the necessary Maven environment variables. Add them to your profile or rc file for persistence.

   ```
   $ export M2_HOME=/usr/local/Cellar/maven/<version_here>
   $ export M2=$M2_HOME/bin
   $ export PATH=$M2:$PATH
   ```

4. Verify proper installation and environment variables with

   ```
   $ mvn -v
   ```

5. Change into the PolyFold directory containing `pom.xml`

6. Compile

   `$ mvn clean javafx:jlink`

7. Run

   `$ ./target/PolyFold/bin/launcher`

Debian Based Linux

1. Install Maven

   `$ sudo apt install maven`

2. Export the necessary Maven environment variables. Add them to your profile or rc file for persistence.

   ```
   $ export M2_HOME=/usr/share/maven/
   $ export M2=$M2_HOME/bin
   $ export PATH=$M2:$PATH
   ```

3. Verify proper installation and environment variables with

   `$ mvn -v`

4. Change into the PolyFold directory containing `pom.xml`
5. Compile

   `$ mvn clean javafx:jlink`

6. Run

   `$ ./target/PolyFold/bin/launcher`

Windows

If you wish to install PolyFold from source on Windows it is recommended that you do so on a 64 bit Windows 10 installation using the IntelliJ IDE.

1. Ensure that the `JAVA_HOME` and `JAVAFX_HOME` environment variables are set properly under `Settings > Path Variables`.
2. Ensure that the Maven plugin suite is installed in IntelliJ.
3. Open the PolyFold project in IntelliJ (the directory containing `pom.xml` is the project directory.
4. Click the Maven plugin tab on the right-hand side of IntelliJ.
5. Navigate the directory hierarchy to:

   `Plugins > JavaFX > javafx:jlink`

6. Double click `javafx:jlink`. This will trigger the build process.
7. Run `target\PolyFold\bin\launcher.bat` to launch PolyFold.

2.4 License

PolyFold is distributed under the **GNU General Public License, version 3**. A short, non-exhaustive summary of what this license entails is provided below.

Permissions

- Commercial Use
- Modification
- Distribution
- Patent Use
- Private Use

Limitations

- No Liability
- No Warranty

Conditions

- Notice of License and Copyright
- Disclose Source Code
- Distribute Under Same License

The full terms and conditions of the license may be found at

https://www.gnu.org/licenses/#GPL

3 Features

3.1 Protein Visualization & Manipulation

PolyFold allows users to visualize protein folding in two ways. First, PolyFold renders the 3D structure in a simplistic sphere and rod form in its main view port. This sphere and rod representation is updated in real time as the user modifies the angles between residues. Each residue i describes two angles, θ and τ. The angle $\theta \in [0, \pi]$ is the planar angle between residues $i-1$, i, and $i+1$. In a protein with N residues, residues 1 and N will not have a planar angle and are assigned the dummy planar angle of 2π. The angle $\tau \in [-\pi, \pi]$ is the dihedral angle between the planes A and B, where plane A is described by residues $i-1$, i, and $i+1$, and plane B is described by residues i, $i+1$, and $i+2$. Residues 1, $N-1$, and N will not have a dihedral angle and are assigned the dummy dihedral angle of 2π.

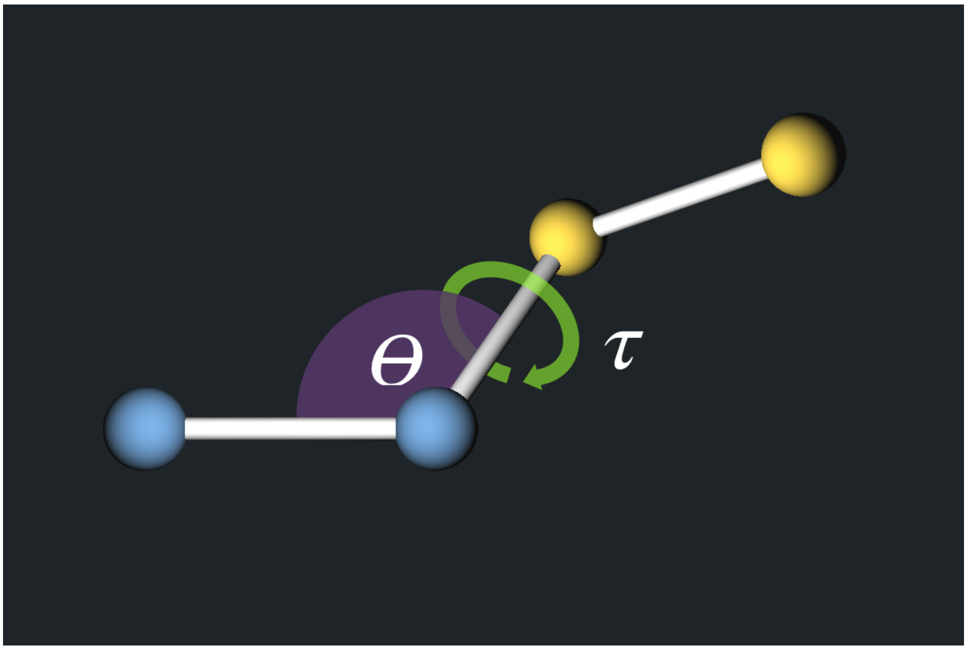

Figure 3.1: a visual representation of planar angle θ and dihedral angle τ

Second, PolyFold generates a modified distance map and displays it in real time as well. The modified distance map is a 2D square matrix displaying different information in its lower and upper triangles. The upper triangle (all cells i, j where $i < j$) color codes the actual distances provided in the loaded Residue-Residue file. The lower triangle (all cells i, j where $i > j$) color codes the current distances of the user manipulated protein. Note that the main diagonal of the distance map contains only zero values.

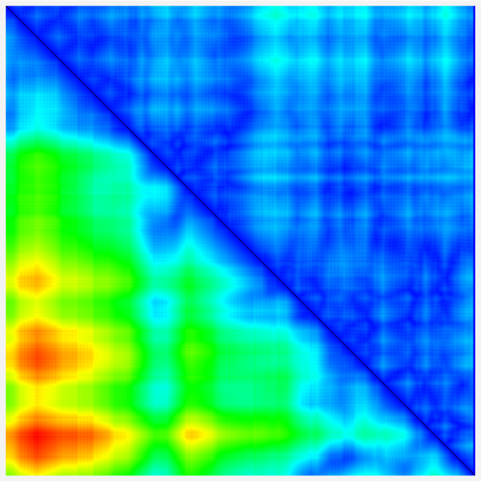

Figure 3.2: an example of the modified distance map

PolyFold not only allows users to dynamically visualize protein structures, it also allows them to manipulate proteins by hand. This is achieved through PolyFold's "Movement" panel, which lets users modify the planar and dihedral angles between subsets of residues.

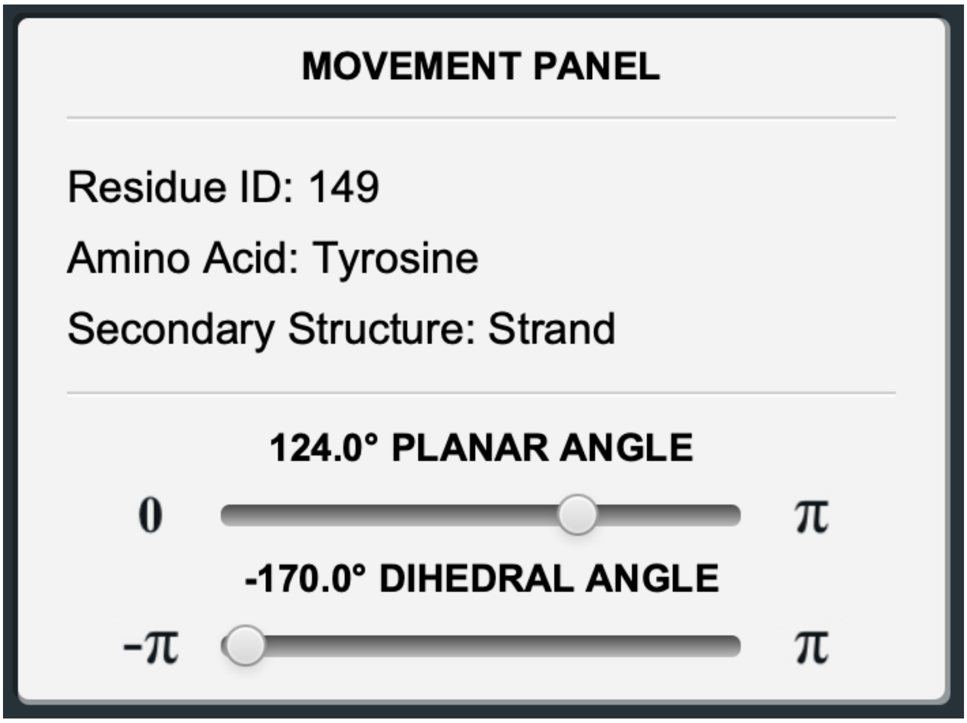

Figure 3.3: the movement panel

3.2 History & Save States

History

PolyFold keeps track of all changes that a user makes to the current protein's fold. The state history will grow indefinitely until the user performs one of four destructive actions:

- loading a new protein structure
- loading a save state ("Quick" or "Named")
- extending the current protein structure (see Extend Structure under Toolbar)
- closing the current session or quitting the application

The state history allows the user to undo and redo any changes they have made to the current protein. However, each new move has the potential to delete states from the state history if a new "time-line" results. This effect is visualized in Figure 3.4.

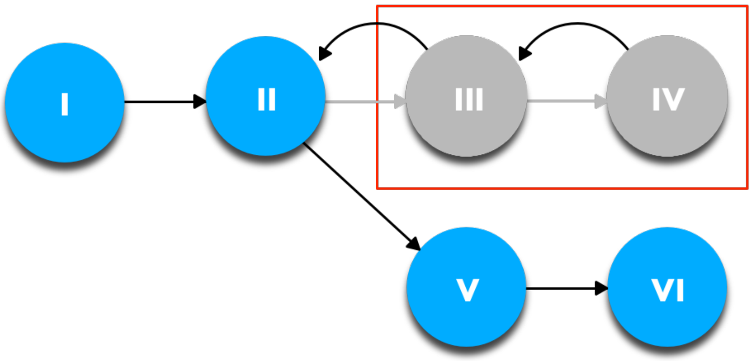

Figure 3.4: states III and IV are lost when move V is applied

Quick Save States

PolyFold's "quick" save states have no names and have a limited capacity. There are exactly 10 quick save states, 0-9, which correspond to the 10 numeric keys on your keyboard.

Named Save States

PolyFold's "named" save states have unique names and unlimited capacity. Creating a named save state with the same name as a pre-existing save state will overwrite it.

3.3 Optimization

PolyFold offers two protein folding optimizations, a Batch Gradient Descent Algorithm [4] (BGDA hereafter) and a Simulated Annealing Algorithm [1] (SAA hereafter). The BGDA is a deterministic algorithm (for a given start state) which translates the currently rendered protein from polar space into Cartesian space and optimizes with respect to the partials of x, y, and z. The SAA is a stochastic algorithm which acts directly on the polar space.

3.3.1 Batch Gradient Descent

Configuration

The BGDA has two configurable parameters — number of iterations and step size.

The number of iterations will determine how long the algorithm runs. Setting the number of iterations too small for larger proteins may leave the protein in only a partially optimized state, while setting the number of iterations too large will result in much longer run-times. Performing BGDA on larger proteins will have a naturally longer runtime than smaller proteins regardless of the number of iterations. This is because the amount of computation per iteration of PolyFold's BGDA grows with $O(N^2)$ where N is the number of residues in the protein.

The step size scales the size of the gradient before it is used to shift a residue. Setting the step size to a small value will allow the BGDA to make finer, more precise movements but will increase the amount of time it takes to converge on a local minimum. Setting the step size to a large value will cause the BGDA to make more coarse movements and potentially converge to a reasonable local minimum faster. However, setting the step size to an excessively large value will cause the BGDA to skip wildly around the search space and potentially even escape it completely. PolyFold normalizes each movement which is applied in order to maintain a predefined bond length between adjacent residues, so this “wild” behavior will not cause any problems. It is worth noting, however, that larger proteins are more prone to this phenomena and will accordingly require smaller step sizes in order for the BGDA to work properly.

Implementation

As with all gradient based optimizations, we begin with a function which we are trying to optimize. This is typically referred to as the loss function. PolyFold's loss function [3] is defined as

$$L(x,y,z) = \begin{cases} (r-l)^2 & r < l \\ 0 & l \le r \le u \\ (r-u)^2 & u < r \le u + 0.5 \\ r - (u + 0.5) + 0.25 & r > u + 0.5 \end{cases}$$

Here, r denotes the distance between two residues and thus can be substituted with $\sqrt{x^2 + y^2 + z^2}$. The terms l and u represent the lower and upper bounds of the possible distances between the current residue pair.

The partial derivatives of x for each of the four sub-functions can be calculated as

$$\frac{\partial L}{\partial x} = \begin{cases} 2x - \frac{2xl}{r} & r < l \\ 0 & l \leq r \leq u \\ 2x - \frac{2xu}{r} & u < r \leq u + 0.5 \\ \frac{x}{r} & r > u + 0.5 \end{cases}$$

The partial derivatives for y and z are identical and obtained simply by substituting for x.

The pseudo code for PolyFold's implementation of BGDA follows in Algorithm 1.

Algorithm 1 Batch Gradient Descent

```
procedure BGDA(residues)                          ▷ residues contains Cartesian points
    for n = 1 up to iterations do
        for i = 1 up to proteinLength do
            gradientSum ← [0, 0, 0]
            for j = i + 1 up to proteinLength do
                r ← distance(residues[i], residues[j])
                if r < l then
                    gradient ← partial1(x, y, z)      ▷ x, y, z are the target coordinates
                else if l ≤ r and r ≤ u then
                    gradient ← [0, 0, 0]              ▷ partial 2 is always 0̂
                else if u < r and r ≤ u + 0.5 then
                    gradient ← partial3(x, y, z)
                else
                    gradient ← partial4(x, y, z)
                end if
                gradientSum = gradientSum + gradient
                residues[j] = residues[j] − stepSize · gradientSum
            end for
        end for
    end for
end procedure
```

Suggested Usage

It is recommended that PolyFold's BGDA is used with a relatively low step size (e.g. $\alpha < 0.01$) and smaller numbers of iterations per run as the protein size increases. The BGDA is a powerful tool, but it is limited by its start state. Whatever local minimum is nearest to the start state is what PolyFold's BGDA will find (though a future version may

implement momentum to mitigate this shortcoming). Therefore, it is recommended to use a combination of SAA runs and BGDA runs to somewhat randomize the start states that the BGDA will optimize.

3.3.2 Simulated Annealing

Configuration

The SAA has four configurable parameters — seed, decay rate, start temperature, and stop temperature.

The seed parameter is a means for recovering determinism within the SAA. Repeated runs which start from the same start state and are initialized with the same seed will yield the same results.

The decay rate controls how quickly the temperature of the SAA will decline. It is simply a factor $\in (0, 1)$ which is multiplied with the temperature at each decay level. Thus, the temperature follows a geometric sequence where $T_{k+1} = \alpha \cdot T_k$. This sequence S can be expressed in terms of a starting temperature T_0 as

$$S_{i=0}^{k} = \alpha^k \cdot T_0$$

Setting a very high decay rate (e.g. $\alpha = 0.99$) will cause the SAA to very slowly approach the final temperature, yielding a longer runtime. Setting a very low decay rate (e.g. $\alpha = 0.01$) will cause the SAA to rapidly approach its final temperature, yielding a shorter more volatile runtime.

The start temperature sets the initial temperature of the SAA. It is a value $\in [0, 1]$. The higher the temperature is, the more likely it is that the SAA will accept a worse state at the current moment in time. So, setting a high start temperature allows more randomness into the SAA. This is not necessarily a bad thing, as long as the final temperature is not also high. Setting a low start temperature will cause the algorithm to perform less randomly and more similar to a greedy algorithm, only accepting better states.

The stop temperature sets the temperature below which the SAA will terminate. It is a value $\in (0, 1]$. Setting a high final temperature is typically ill-advised. This will cause the SAA to perform almost randomly. This is inefficient and doesn't yield any notable results usually. Setting a low final temperature allows the SAA to become more and more greedy over time, which is usually desirable.

At each iteration, the SAA perturbs the planar and dihedral angles of exactly one residue, chosen at random. For each given secondary structure, whether coils, α helices, or β strands, PolyFold defines a magnitude of perturbation for the planar and dihedral angles for residues of that secondary structure. Each of these magnitudes, Δ, are defined in Table 1. The actual perturbation for each angle is chosen randomly from the interval $[-\Delta, \Delta]$ and then scaled by the current temperature, which is a real number in the interval $[0, 1]$. Certain perturbations are disallowed — namely those that cause the planar or dihedral angles to exceed there known bounds [2] defined in Table 2.

Table 1 - SAA Angle Δ per Secondary Structure

Secondary Structure	Angle Δ
Coil	$10°$
α Helix	$6°$
β Strand	$4°$

Table 2 - Secondary Structure Angle Bounds

Secondary Structure	Planar Viable Interval	Dihedral Viable Interval
Coil	$[0°, 180°]$	$[-90°, 90°]$
α Helix	$[77°, 101°]$	$[30°, 70°]$
β Strand	$[110°, 138°]$	$[145°, 235°]$

You may have noticed that the number of iterations is not a configurable parameter. This is because the SAA does not necessarily benefit from having more iterations. Due to the random nature of the SAA, more iterations will only benefit the SAA if they occur at just the right times (e.g. when the SAA is exploring unpromising parts of the search space). Instead, the SAA benefits more strongly from having the right cooling schedule, as defined by the parameters above.

Implementation

The SAA is a fairly traditional implementation. It has all the common components — the concept of energy levels, an acceptance probability, a temperature, and a cooling schedule. The energy level is calculated via the same loss function [3] that we used in the BGDA. The acceptance probability function is

$$P(x'|x) = \begin{cases} 1 & x' < x \\ exp\left(\dfrac{x - x'}{t}\right) & x' \geq x \end{cases}$$

Here, x is the current energy, x' is the energy of the candidate state, and t is the current temperature.

The pseudo code for PolyFold's Implementation of SAA follows in Algorithm 2.

Suggested Usage

It is recommended that PolyFold's SAA is used with a high decay rate (e.g. $\alpha \geq 0.8$), a high start temperature (e.g. $T_0 = 1$), and a low stop temperature (e.g. $T_f \approx 0$). Due to the random nature of the SAA, it is not recommended that it is run prior to any BGDA runs. The BGDA can be conceptualized as climbing the nearest hill, and the SAA can be conceptualized as finding the right hill to climb.

Algorithm 2 Simulated Annealing

procedure $SAA(state)$
  $temperature \leftarrow startTemperature$
  $lowestEnergy \leftarrow getEnergy(state)$
  while $temperature > stopTemperature$ **do**
    for $n = 1$ up to $decayLevelIterations$ **do**
      $energy \leftarrow getEnergy(state)$
      $candidate \leftarrow getRandomNeighbor(state)$
      $energy' \leftarrow getEnergy(candidate)$
      if $energy' < energy$ or $random() < acceptance(lowestEnergy, energy)$ **then**
        $state \leftarrow candidate$
        if $energy' < lowestEnergy$ **then**
          $lowestEnergy \leftarrow energy'$
        end if
      end if
    end for
    $temperature = temperature \cdot decayRate$
  end while
end procedure

3.4 Performance

PolyFold is designed to be responsive and efficient. All optimizations should be able to run in real time without a problem. With that said, the complexity of the BGDA greatly increases as the size of the protein grows. For this reason, there is a size restriction of 500 residues for which proteins PolyFold is able to load and render.

3.5 Menus

File Menu

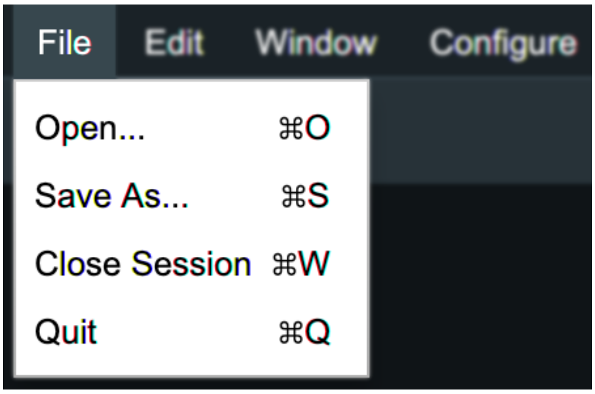

- Open - prompts the user to load a Residue-Residue (.rr) file and begin working on a new protein structure

- Save As... - prompts the user to name and save the currently rendered protein structure to Protein Data Bank (.pdb) format
- Close Session - ends the current protein folding session, removing the current protein from view and returning to PolyFold's landing screen
- Quit - closes the application

Edit Menu

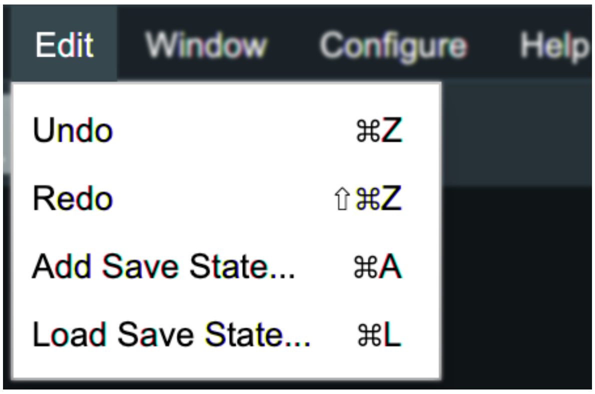

- Undo - revert the previous movement applied to the protein structure
- Redo - re-apply an undone movement in the undo history
- Add Save State... - prompts the user to add a named save state
- Load Save State... - prompts the user to load a named save state

Window Menu

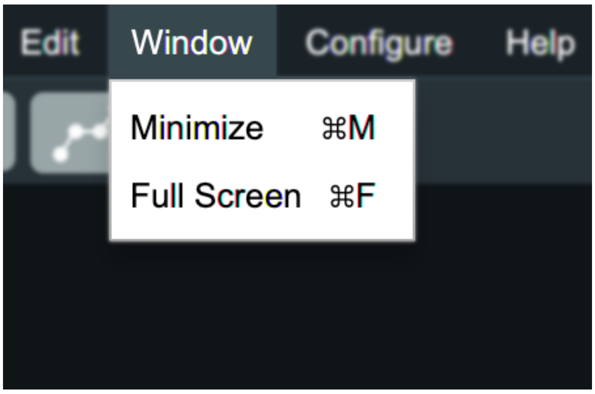

- Minimize - hide the current window in the system dock or tray
- Fullscreen - resize the window to the screen size and hide the status bar

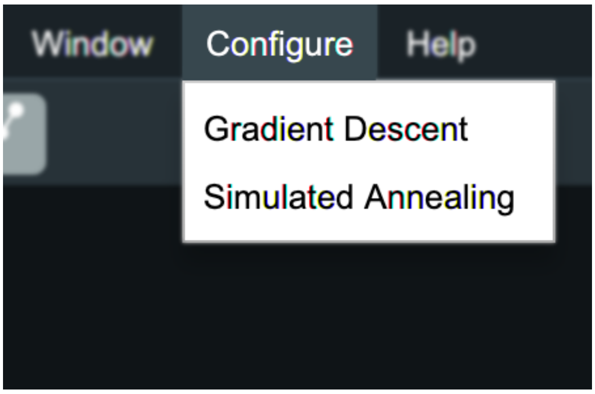

Configure Menu

- Gradient Descent - configure the parameters of the Batch Gradient Descent algorithm
- Simulated Annealing - configure the parameters of the Simulated Annealing algorithm

Help Menu

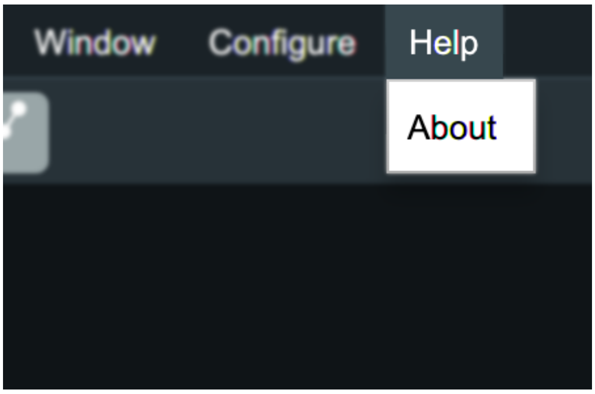

- About - view information about the developers, copyright & license, and the website

3.6 Toolbar

Auto Zoom Button

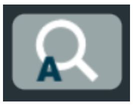

The auto zoom button attempts to move the camera such that the majority of the current protein is visible in the main view port. It is a toggle button and is green when active.

Extend Structure Button

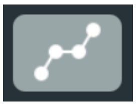

The extend structure button acts as a reset button during a protein folding session. It will re-initialize the protein structure based on the idealized angles of its secondary structure. The moves applied by this button are destructive (e.g. they clear the state history).

Gradient Descent Button

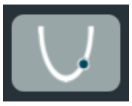

The gradient descent buttons will begin PolyFold's Batch Gradient Descent algorithm. A red cancel button will appear during its execution, allowing you to cancel the optimization at any point in time. The escape key on your keyboard has the same effect.

Simulated Annealing Button

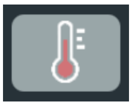

The Simulated Annealing button will begin PolyFold's Simulated Annealing algorithm. A red cancel button will appear during its execution, allowing you to cancel the optimization at any point in time. The escape key on your keyboard has the same effect.

Undo Button

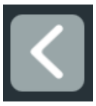

The undo button will revert the previous movement applied to the protein structure.

Redo Button

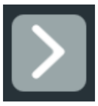

The redo button will re-apply an undone movement in the undo history.

Info Button

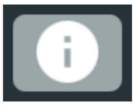

The info button will open a dialog containing information about the developer, copyright & license, and the website.

4 Usage

4.1 File Formats

Input Files

PolyFold works with Residue-Residue (extension .rr) files in order to build and visualize protein structures in 3D. PolyFold anticipates that its users will adhere to the PFRMAT RR format detailed at

http://predictioncenter.org/casp13/index.cgi?page=format#RR

However, PolyFold does not enforce that the file is perfectly formatted, merely that all the information it needs to function is provided. Specifically PolyFold expects the file to follow:

- Line 1
  The amino acid sequence string terminated by a new line
- Line 2
  The secondary structure string terminated by a new line; the length of this line must match the length of Line 1
- Lines 3+
  Several lines following the format

  $$i\ j\ d_1\ d_2\ p$$

  where N is the length of the amino acid sequence, i is the ith residue, j is the jth residue, d_1 is the lower bound distance between residues i and j, d_2 is the upper bound distance between residues i and j, and p is a real number indicating the probability of the inter-residue interaction. As such, PolyFold will ignore p and instead assume $p = 1$ for all interacting pairs. As an aside, the word "several" is simply used to acknowledge that PolyFold allows for a slightly flexible format. However, the exact number of lines required is equivalent to the number of residue pairs (i, j) which satisfy $i < j$. This can be formally calculated as

  $$\sum_{i=1}^{N-1} i$$

  for a protein with N residues. So, PolyFold checks that each such residue pair exists and is defined properly and warns the user of an improperly formatted Residue-Residue file otherwise.

Output Files

PolyFold has the capability of saving the currently rendered protein structure to a correctly formatted Protein Data Bank (extension .pdb) file. More information about the PDB format can be found at

http://wwpdb.org/documentation/file-format-content/format33/

4.2 Keyboard Shortcuts

The following table exhaustively defines all of PolyFold's keyboard shortcuts. Please note the use of the word "Super" denotes the Command key on Mac machines and the Control key on most other machines, and the word "Alt" denotes the Option key on Mac machine and the Alt key on most other machines.

**Action**	**Shortcut**
Open File	Super + O
Save File As	Super + S
Close Session	Super + W
Quit Application	Super + Q
Undo	Super + Z
Redo	Super + Shift + Z
Add Quick Save State [0-9]	Super + Alt + [0-9]
Load Quick Save State [0-9]	Super + [0-9]
Add Named Save State	Super + A
Load Named Save State	Super + L
Minimize Window	Super + M
Fullscreen Window	Super + F
Cancel Optimization	Escape

5 Modifying PolyFold

Since PolyFold is distributed under the open source GNU GPL v. 3 license, you are free to modify its source code to adapt it to your own needs. The code is mostly documented in comments, but is constantly growing and changing. To aid in your development the code has been separated into functional modules. Figure 5.1 describes PolyFold's modules and classes.

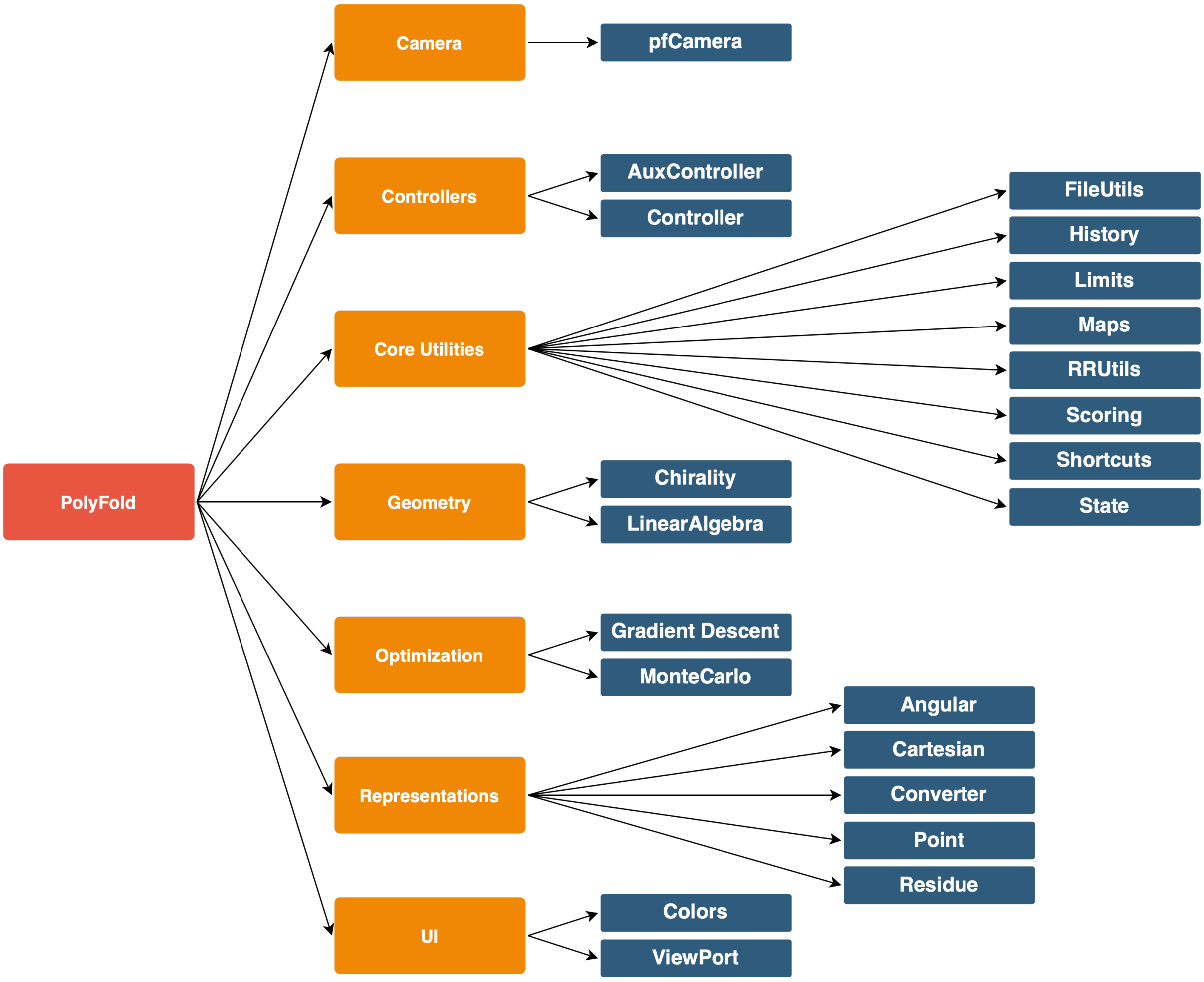

Figure 5.1: PolyFold's module and class structure

High level descriptions of each module are as follows.

- camera - a camera class extending the JavaFX PerspectiveCamera for PolyFold's needs
- controllers - a primary and auxiliary controller for handling events
- coreutils - essential functions of PolyFold including file I/O, history, scoring, etc.
- geometry - PolyFold's geometry engine for rendering 3D proteins
- optimizations - the batch gradient descent and simulated annealing algorithms
- representations - classes for representing residues in Cartesian and polar spaces
- ui - classes containing color information and PolyFold's 3D view port class

6 Acknowledgments

First, I'd like to acknowledge my advisor, Dr. Debswapna Bhattacharya. Without his belief in me and in this project, PolyFold would not exist. As I was writing this manual, I looked back in our GitHub history to see how PolyFold started, and it is absolutely insane how far it has come. You've been a huge inspiration and role model for me in the type of academic that I would like to become. You are so open-minded and intellectually humble, and it's a truly exceptional quality for someone as smart and hard working as you are. Thank you for your investment in my life and work.

Further, I'd like to acknowledge Rahmatullah Roche and Sutanu Bhattacharya, two graduate students who helped curate the initial datasets for PolyFold's development and who played an integral role in testing it. You both are great students and workers, and I look forward to the outstanding work that you will contribute in your academic careers. I'd also like to acknowledge Hudson Chromy and Carmen Stowe for the helpful discussions and feedback that they offered in the early stages of PolyFold's development.

Finally, I'd like to acknowledge my school — Auburn University. I feel deeply grateful and consider it a privilege to study here. I'm constantly surrounded by people who are pursuing academic excellence, and they sharpen me on a daily basis. So, if you are an Auburn professor, alumni, or student and you are reading this, thank you and War Eagle!

Bibliography

[1] E. Aarts and J. Korst. “Simulated annealing and boltzmann machines”. In: (Jan. 1988).

[2] G. Labesse et al. “P-SEA: a new efficient assignment of secondary structure from Cα trace of proteins”. In: *Bioinformatics* 13.3 (June 1997), pp. 291–295. ISSN: 1367-4803. DOI: 10.1093/bioinformatics/13.3.291. eprint: https://academic.oup.com/bioinformatics/article-pdf/13/3/291/1170655/13-3-291.pdf. URL: https://doi.org/10.1093/bioinformatics/13.3.291.

[3] Andrew Leaver-Fay et al. “Chapter nineteen - Rosetta3: An Object-Oriented Software Suite for the Simulation and Design of Macromolecules”. In: Methods in Enzymology 487 (2011). Ed. by Michael L. Johnson and Ludwig Brand, pp. 545–574. ISSN: 0076-6879. DOI: https://doi.org/10.1016/B978-0-12-381270-4.00019-6. URL: http://www.sciencedirect.com/science/article/pii/B9780123812704000196.

[4] Tong Zhang. “Solving Large Scale Linear Prediction Problems Using Stochastic Gradient Descent Algorithms”. In: ICML ’04 (2004), p. 116. DOI: 10.1145/1015330.1015332. URL: https://doi.org/10.1145/1015330.1015332.
